# Data on the effect of electrospinning parameters on the morphology of the nanofibrous poly(3-hydroxybutyrate-*co*-4-hydroxybutyrate) scaffolds

**DOI:** 10.1016/j.dib.2019.104777

**Published:** 2019-11-11

**Authors:** C.J. Chai, A.A. Amirul, S. Vigneswari

**Affiliations:** a Faculty of Science and Marine Environment, Universiti Malaysia Terengganu, Malaysia; b School of Biological Sciences, Universiti Sains Malaysia, Penang, Malaysia

**Keywords:** Polyhydroxyalkanotes (PHAs), poly(3-hydroxybutyrate-*co*-4-hydroxybutyrate) [P(3HB-*co*-4HB)], Electrospinning, Nanofibers, Bacterial polymer

## Abstract

Electrospinning is a promising approach to fabricate desirable electropsun nanofibrous scaffold that could be applied in the medical fields. In this study, bacterial copolymer poly(3-hydroxybutyrate-*co-*68 mol% 4-hydroxybutyrate) [P(3HB-*co*-68mol% 4HB)] copolymer produced was fabricated into electrospun nanofibers using various combination of electrospinning parameters including the polymer solution, applied voltage and injection speed. The morphology of the fabricated scaffolds were observed using scanning electron microscope (SEM). The SEM images were analysed for the fibre diameter distribution of the scaffolds using Image Analyser. The results revealed that the 8 wt% of polymer solution, 25 kV/cm of the applied voltage and 1.5 mL/h of the injection speed was the most suitable combination. This electrospinning parameters combination fabricated nanofibrous P(3HB-*co*-4HB) scaffold with smooth, beadles and uniform nanofibers with small fibre diameter distribution.

**Table:** Specifications Table

Subject	Materials Science
Specific subject area	P(3HB-*co*-4HB) Electrospun nanofibers
Type of data	Raw data, tables and figures
How data were acquired	The images were acquired by scanning electron microscope (SEM) and the SEM images were interpreted by Image Analyser to acquire the fibre diameter distribution.
Data format	Raw and analysed
Parameters for data collection	The polymer solution, applied voltage and injection speed during the electrospinning process are the major parameters for data collection.
Description of data collection	The electrospun nanofibrous scaffolds fabricated by different combination of parameters were observed under SEM. The SEM images were then taken to analyse the fibre diameter of the electrospun nanofibers on the scaffold by using Image Analyser.
Data source location	Malaysian Institute of Pharmaceuticals and Nutraceuticals (Ipharm), Gelugor, Pulau Pinang, Malaysia and Universiti Malaysia Terengganu, Kuala Nerus, Terengganu, Malaysia
Data accessibility	The data are incorporated within this article.

**Table:** 

**Value of the Data** • The data can provide useful information for other groups working on the fabrication of smooth and beadless nanofibers by optimising the electrospinning parameters. • The data are useful to develop an optimum electrospinning method for development of electrospun nanofiber materials using bacterial polymer. • The data is valuable as it provides basic information that could be used to develop desirable nanofibrous scaffold for various biomedical purposes.

## 1 Data

**Fig. 1:**
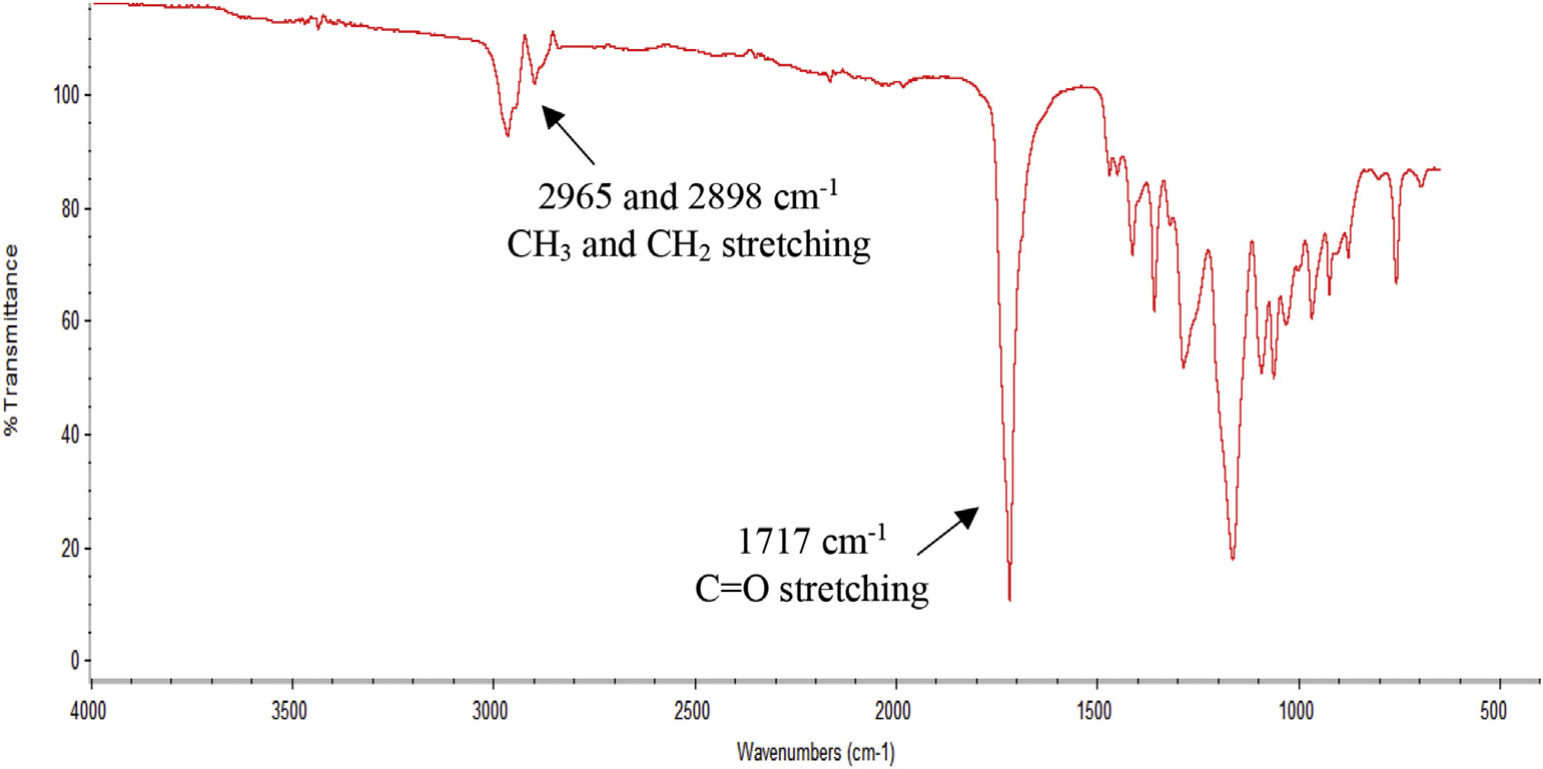
FTIR spectrum of P(3HB-*co*-4HB) polymer.

The data presented are focused on the morphology of the P(3HB-*co*-4HB) nanofibers and their fibre diameter distributions fabricated using electrospinning. Table 1 summarizes the SEM images and fibre diameter distribution of the scaffolds under different combination of the parameters during electrospinning process. The concentration of the polymer solution, applied voltage and injection speed are the parameters that can be manipulated to develop the smooth, uniform and beadless nanofibers. The chemical functional groups of the P(3HB-co-4HB) scaffolds using fourier transform infrared spectroscopy (FTIR) were shown in Fig. 1. Thermal behaviours of the P(3HB-co-4HB) copolymer were determined using differential scanning calorimetry and summarised in Table 2.

**Table 1:** SEM images and the fibre diameter distribution of the P(3HB-*co*-4HB) scaffolds under different electrospinning parameters.

Scaffold	Parameters used and SEM image	Fibre diameter distribution
A	• 6% w/v polymer solution • Voltage: 22 kV • Injection speed: 1.5 mL/h	
B	• 6% w/v polymer solution • Voltage: 25 kV • Injection speed: 1.5 mL/h	
C	• 7% w/v polymer solution • Voltage: 25 kV • Injection speed: 1.5 mL/h	
D	• 8% w/v polymer solution • Voltage: 25 kV • Injection speed: 1.5 mL/h	
E	• 8% w/v polymer solution • Voltage: 25 kV • Injection speed: 1.4 mL/h	
F	• 8% w/v polymer solution • Voltage: 25 kV • Injection speed: 1.6 mL/h	
G	• 9% w/v polymer solution • Voltage: 25 kV • Injection speed: 1.5 mL/h

**Table 2:** Thermal properties of P(3HB-*co*-4HB) polymer.

Scaffold	Glass transition temperature, *T*_g_ (^o^C)	Crystallization temperature, *T*_c_ (^o^C)	Melting temperature, *T*_m_ (^o^C)	Heat of fusion, Δ*H*_m_ (J/g)
P(3HB-*co*-4HB)	−42.26 ± 0.19	3.02 ± 3.62	58.67 ± 0.37	35.499 ± 2.386

## 2 Experimental design, materials, and methods

### 2.1 Biosynthesis of P(3HB-*co*-4HB)

The production of copolymer P(3HB-*co*-4HB) was conducted in a two-stage cultivation process in 15L bioreactor with 10L working volume. The microorganisms *Cupriavidusmalaysiensis* USMAA1020 transformant were grown in nutrient-rich (NR) broth (10 g of peptone, 10 g of lab-lemco powder and 2 g of yeast extract in 1 L distilled water) for 12 hours and then transferred 0.1 g/L of the bacteria culture into nitrogen-free mineral salts medium (MSM) [1]. The mixture of both 1,4-butanediol and 1,6-hexanediol were used as the 4HB carbon precursors for the biosynthesis [2]. After 84 hours cultivation, the cultures were harvested by centrifugation at 10000 rpm for 15 minutes and then freeze-dried.

### 2.2 Analytical procedures

The PHA content and compositions in the lyophilized cells were determined using gas chromatography (GC-17A, Shimadzu, Kyoto, Japan). Based on the GC method with some modification [3], approximately 10 mg of the lyophilized cell was subjected to methanolysis in the presence of methanol and sulphuric acid [85:15% (v/v)]. The organic layer which contained reaction products was dried with Na_2_SO_4_, and analysed by GC.

### 2.3 P(3HB-*co*-4HB) extraction

Approximately 1 g of freeze-dried cells were stirred in 200 mL chloroform at room temperature for 48 hours [1]. The extracts were filtered using filter paper and then the filtrate was concentrated using the rotary evaporator. About 200 mL cold methanol was stirred on a magnetic stirrer while the concentrated solution was added drop-wise to precipitate the dissolved polymer. The precipitated polymer was recovered by filtration using a 0.45 μm PTFE membrane and dried overnight in the fume hood.

### 2.4 Fourier transform infrared spectroscopy (FTIR)

The FTIR spectroscope (PerkinElmer Spectrum GX) was used to analyse the functional groups present in P(3HB-*co*-4HB). The spectra of each sample were obtained in the range of 4000–500 cm^−1^ at a resolution of 4 cm^−1^. The spectral outputs were recorded in transmittance as a function of wave number [4].

### 2.5 Differential scanning calorimetry (DSC)

DSC analysis was performed with a Diamond PerkinElmer Pyris 1 thermal analyser (PerkinElmer Inc., USA) equipped with liquid nitrogen cooling accessory. Approximately 5 mg of samples was encapsulated in aluminium pans and heat from −50 °C to 200 °C at a scanning rate of 10 °C/min (first heating scan). The samples were maintained at 200 °C for 2 min and then rapidly quenched to −50 °C. After maintaining at −50 °C for 5 min, the samples then reheated from −50 °C to 200 °C at a scanning rate of 10 °C/min (second heating scan). The DSC curve from the second heating scan was used to analyse the thermal property. The glass transition temperature (Tg) was taken as the midpoint of the heat capacity change, the crystallization temperature (Tc) was taken at the peak of the enthalpy of exotherm while the melting temperature (Tm) and the enthalpy of fusion (ΔHm) were taken at the peak of the melting endotherm, respectively [5].

### 2.6 Fabrication of electrospun P(3HB-*co*-4HB) nanofibers via electrospinning

The electrospinning process was carried out using a custom-built Nano Fibre Production System (NEU-202) instrument. The polymer solution was prepared by dissolving P(3HB-*co*-4HB) in mixed solvent of dimethylformamide (DMF) and chloroform (v/v) prepared at a ratio of 1:4. The polymer solution was loaded in 5 mL syringes with metal blunt needle of 21 gauge (G) and diameter of 10 mm. The X-axis was automated sequenced at a speed of 10 mm/s starting from 140 mm to 165 mm while Y-axis was set at 195 mm. The polymer solution was extruded using a computer controlled syringe pump and subjected to an electric potential to fabricate the electrospun nanofibers. The electrospun nanofibers were collected on a collecting plate at a working distance of 10 cm perpendicular to the needle tip. The concentration of the polymer solution, applied voltage and injection speed were adjusted during electrospinning process. The temperature was regulated at 25 ± 2 °C with relative humidity of 28 ± 2% [4].
