# Supplementary figures and images for: Data on the effect of electrospinning parameters on the morphology of the nanofibrous poly(3-hydroxybutyrate-co-4-hydroxybutyrate) scaffolds
Source: Data Brief. 2019 Nov 11;28:104777. doi: 10.1016/j.dib.2019.104777 (PMC6911942; doi:10.1016/j.dib.2019.104777)

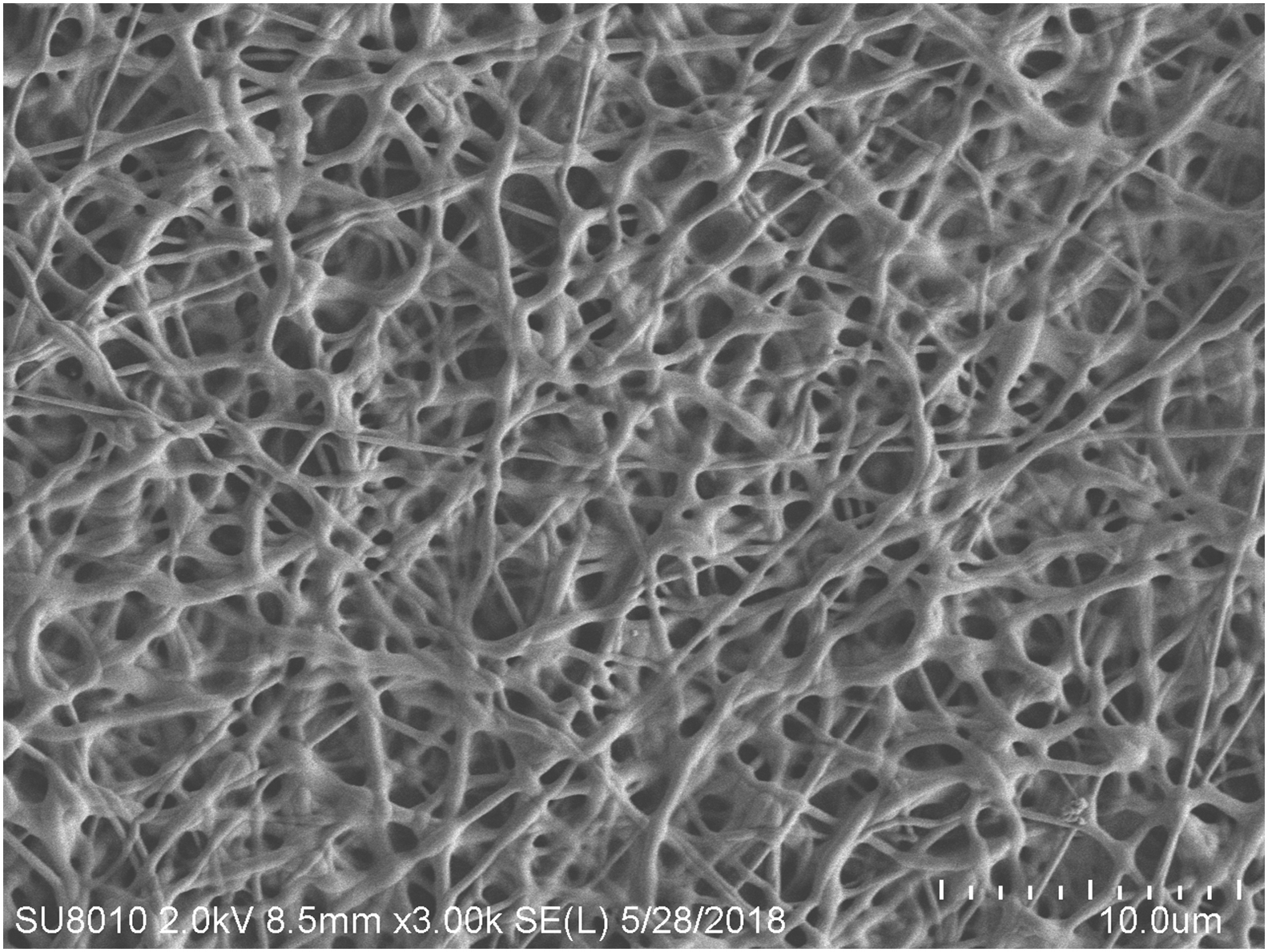

Supplement: Multimedia component 1 [file figs1.jpg]

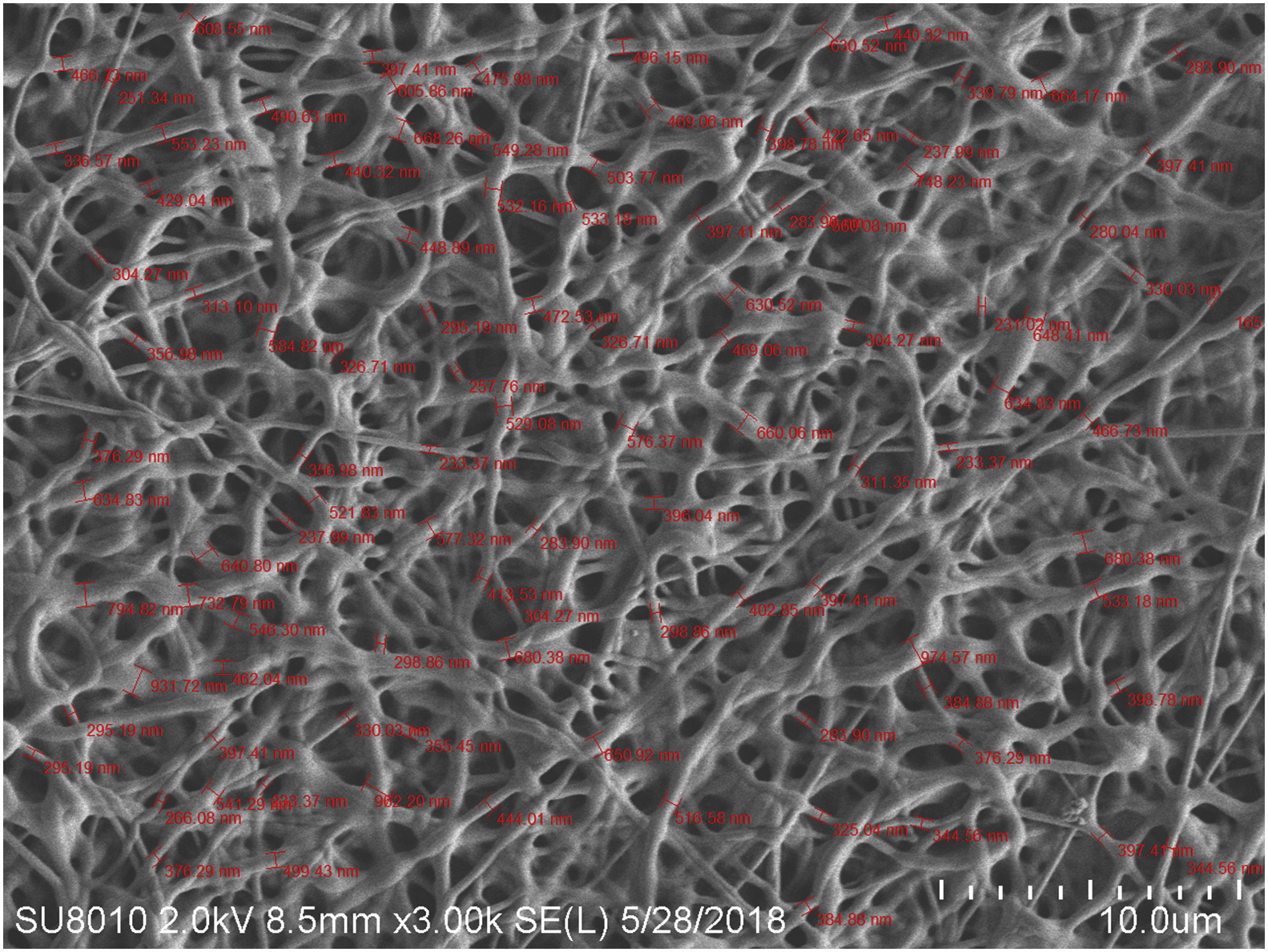

Supplement: Multimedia component 2 [file figs2.jpg]
